# Mesenchymal Stem Cells in Veterinary Medicine—Still Untapped Potential

**DOI:** 10.3390/ani15081175

**Published:** 2025-04-19

**Authors:** Magdalena Morawska-Kozłowska, Mateusz Pitas, Yauheni Zhalniarovich

**Affiliations:** 1 Department of Surgery and Radiology with Clinic, Faculty of Veterinary Medicine, University of Warmia and Mazury in Olsztyn, 10-719 Olsztyn, Poland; eugeniusz.zolnierowicz@uwm.edu.pl; 2 Veterinary Polyclinic, University of Warmia and Mazury in Olsztyn, 10-719 Olsztyn, Poland; pitasmateusz95@gmail.com

**Keywords:** stem cells, stem cell therapy, MSC, veterinary regenerative medicine

## Abstract

Mesenchymal stem cells (MSCs) hold significant therapeutic potential in veterinary medicine due to their regenerative and immunomodulatory properties. This review examines the clinical applications of MSCs across multiple animal species, including equine, canine, feline, and bovine medicine. MSC therapies have demonstrated promising outcomes in treating musculoskeletal disorders, osteoarthritis, inflammatory diseases, and tissue injuries, particularly in horses and dogs. In cats, MSCs show potential for managing chronic kidney disease, inflammatory bowel disease, and asthma, while in bovine medicine, they offer alternative treatment approaches for mastitis and orthopedic injuries. Despite these advancements, challenges such as treatment standardization, cell sourcing, and potential adverse effects, including tumorigenicity, remain under investigation. The emerging field of MSC-based veterinary medicine highlights its capacity to enhance healing, reduce inflammation, and improve clinical outcomes. However, further research is necessary to optimize treatment protocols and address safety concerns, ensuring the widespread adoption of MSC therapies in veterinary practice.

## Simple Summary

Mesenchymal stem cells (MSCs) are a type of adult stem cell that can be found in various tissues such as bone marrow, adipose tissue, umbilical cord, and dental pulp. These cells have remarkable abilities to self-renew and differentiate into a wide range of cell types. Due to their versatile properties, MSCs are gaining increasing recognition and use in veterinary medicine, with numerous potential applications. This article provides a comprehensive literature review of the clinical uses of MSCs within the field of veterinary medicine.

## 1. Introduction

**Figure 1:**
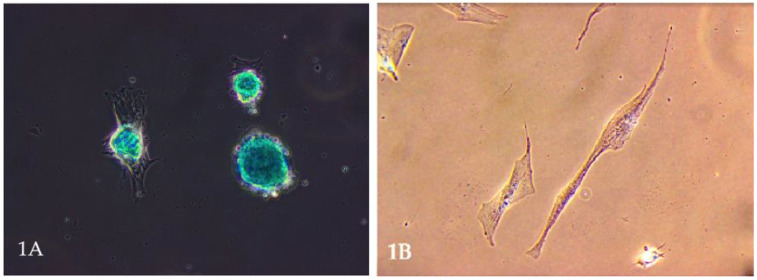
(**A**). Microscopic image of stem cell differentiation into a chondrocyte population. The picture shows a chondrocyte monolayer. Own research. (**B**). Mesenchymal stem cells are obtained from a bitch uterus. Own research.

Mesenchymal stem cells (MSCs) have garnered significant attention in veterinary medicine. These multipotent stromal cells, capable of differentiating into various cell types, including osteocytes, chondrocytes (Figure 1A), and adipocytes, present promising therapeutic avenues for various animal diseases [1,2]. Their ease of isolation from tissues such as bone marrow, adipose tissue, umbilical cord, and even the uterus (Figure 1B), coupled with minimal ethical concerns, further enhances their appeal in clinical settings [3,4].

In recent years, the application of MSCs in veterinary practice has expanded, particularly in treating musculoskeletal disorders in companion animals like dogs and horses. Studies have demonstrated that MSC-based therapies can effectively promote tissue repair and modulate inflammatory responses, improving outcomes in conditions such as osteoarthritis and tendon injuries [5]. For instance, research indicates that MSCs can differentiate into chondrocytes, contributing to cartilage regeneration in degenerative joint diseases [6,7,8]. Beyond musculoskeletal applications, MSCs have shown potential in addressing a variety of other conditions. Their immunomodulatory capabilities make them suitable for treating inflammatory and autoimmune diseases, while their ability to secrete bioactive factors supports tissue healing and regeneration [9]. MSCs have been explored in canine models for their therapeutic effects on skin wounds and neuromuscular disorders, with promising preliminary results [10].

Mesenchymal stem cell therapy shows promise in treating various ocular diseases in veterinary medicine, including corneal ulcers, keratitis, and retinal degeneration in cats, dogs, and horses. MSCs have immunomodulatory and regenerative properties, reducing inflammation and promoting healing. Studies highlight their ability to reduce corneal opacity and vascularization, improving vision [11,12].

Due to the numerous properties of mesenchymal stem cells, it is unsurprising that they are the subject of extensive research by contemporary scientists and pharmaceutical companies. Actually, the first pharmaceutics with MSCs are starting to appear, like DogStem [13]. However, the potential adverse effects of therapies utilizing these cells are rarely discussed. This issue has been addressed by Ghollasi et al., who mention that MSCs may promote tumor growth through their ability to induce angiogenesis [14], suppress immune responses, create a tumor-supportive microenvironment [15], and undergo malignant transformation themselves [16,17]. However, several studies have explored the tumorigenicity of stem cells in immunodeficient mice [18]. In one study, after 40 days of observation, no tumor formation was detected, unlike the positive control group that received HeLa cells [19]. In another study with nude mice, different doses of human adipose-derived MSCs were injected and monitored for 13 weeks following intravenous injection and 26 weeks after subcutaneous injection. No instances of toxicity or tumor formation were observed in any of the dose groups, including the high-dose group [20].

The above article aimed to review the literature on the clinical application of mesenchymal stem cells in veterinary medicine.

## 2. Materials and Methods

The search strategy involved using electronic databases such as PubMed, Web of Science, and ScienceDirect to identify relevant articles published between 2006 and 2024. Carefully chosen search terms encompassed a broad spectrum of relevant topics, including variations of “mesenchymal stem cells in veterinary”, “mesenchymal stem cells in dogs”, “mesenchymal stem cells in cats”, “mesenchymal stem cells in bovines”, “mesenchymal stem cells in horses”, and related terms.

A total of 143 articles were carefully chosen for inclusion in this review based on their relevance and contribution to understanding the role of mesenchymal stem cells in veterinary medicine. Systematic data extraction was performed, emphasizing key aspects such as the clinical application of MSCs in common diseases affecting species like dogs, cats, horses, and cows. Furthermore, articles published before 2006 were excluded due to advancements in regenerative medicine and the high redundancy of data in older studies.

## 3. Therapeutic Potential in Equine Medicine

Stem cell therapy has emerged as a promising treatment option in veterinary medicine, particularly for equine patients [21,22]. Due to their susceptibility to musculoskeletal injuries, especially in athletic disciplines, horses have become ideal candidates for regenerative therapies involving stem cells [23,24]. The potential of stem cells to differentiate into various tissue types and promote tissue repair has revolutionized treatments for conditions that were previously managed with limited success [25,26].

Tendinitis, particularly injuries to the superficial digital flexor tendon (SDFT), is among the most common issues in athletic horses [27,28]. Traditional treatment options, such as rest and anti-inflammatory medications, often lead to incomplete healing and a high risk of re-injury due to scar tissue formation [21,29]. MSCs stimulate collagen production and enhance tissue remodeling, key processes in tendon repair [30]. Stem cells offer a solution by promoting the regeneration of tendon-like tissue rather than scar formation [31]. These cells also help mitigate inflammation by secreting growth factors that aid in the recovery of damaged tissue and restore functional strength to the tendon [32]. Research has shown that horses treated with MSCs for tendonitis exhibit faster recovery times and fewer complications than those treated with conventional corticosteroid injections or platelet-rich plasma (PRP) therapy [33,34]. Furthermore, studies suggest that stem cell therapy may reduce re-injury risk, a significant concern in horses with tendon damage [35]. Stem cells harvested from different tissues have been used in horses to treat tendonitis. In one study, horses treated with MSCs for tendon injuries showed significant improvement, with nearly 90% returning to athletics without injury recurrence after two years. However, this study used stem cells from bone marrow [36]. AD-MSCs have also demonstrated promising results, with horses showing better tendon repair and less scarring than control groups [21]. No data are currently available on mesenchymal cells derived from the uterus.

Osteoarthritis (OA) in horses is a progressive degenerative joint disease that often leads to pain, stiffness, and decreased mobility [37]. Stem cell therapy has emerged as a promising treatment option for managing OA in horses by promoting tissue regeneration and reducing inflammation [38]. Mesenchymal stem cells, typically harvested from adipose tissue or bone marrow, can differentiate into chondrocytes, aiding in repairing damaged cartilage [38,39]. Studies have shown that MSCs can modulate the immune response and reduce the production of inflammatory mediators within the joint, leading to pain relief and functional improvement [39,40]. Clinical trials have demonstrated that stem cell injections into affected joints can provide short-term and long-term benefits, including improved joint function and decreased lameness [41]. However, the full potential of stem cell therapy in horses with OA still requires further investigation to optimize treatment protocols and assess long-term outcomes [42]. Currently, no information is available on mesenchymal cells from the uterus.

One of the most promising applications of endometrial stem cells (ESCs) is their ability to regenerate damaged endometrial tissue. Endometritis and fibrosis are significant causes of infertility in mares, often leading to poor reproductive performance [43]. In horses, endometriosis can manifest as ectopic endometrial tissue within the abdominal cavity or as uterine fibrosis and scarring following repeated cycles of inflammation. This pathological process interferes with normal uterine function, leading to infertility, chronic pain, or recurrent colic. Traditional treatment methods, such as hormonal regulation and surgical interventions, often fall short due to the progressive and recurrent nature of the disease [44,45]. Stem cell therapy offers a regenerative approach that holds promise for mitigating the damage caused by endometriosis. Stem cells, particularly mesenchymal stem cells, have shown significant anti-inflammatory and antifibrotic properties. Mesenchymal stem cells can influence changes within the mare’s uterus, among others; modulate the immune response to reduce chronic inflammation [46]; inhibit fibrosis by altering the extracellular matrix turnover [47]; and promote angiogenesis and tissue repair within the damaged uterine environment [48]. Stem cells can be administered to treat equine endometriosis through several methods. Intrauterine injection directly delivers stem cells into the uterine lumen, enabling targeted action on the endometrial layer and proving particularly effective for uterine scarring [49]. Intravenous infusion offers systemic delivery, providing broader anti-inflammatory and regenerative effects that may address ectopic lesions outside the uterus [50]. Additionally, laparoscopic application allows for the precise placement of stem cells onto abdominal endometrial implants, making it suitable for cases with abdominal lesions [51]. Although clinical studies on stem cell therapy for equine endometriosis are limited, preliminary evidence from related conditions suggests promising outcomes. For instance, intrauterine MSC therapy has successfully treated endometrial fibrosis in mares, improving uterine health and conception rates [52]. These findings emphasize the potential for applying similar strategies in the management of equine endometriosis.

ESCs are being studied for their broader systemic effects, particularly their anti-inflammatory and immunomodulatory properties. These effects could make ESCs suitable for treating autoimmune diseases and systemic inflammatory conditions in horses. Recent studies suggest that MSCs may modulate the immune system by influencing T cell responses and cytokine production, offering therapeutic potential for conditions like equine recurrent uveitis (ERU) and systemic lupus erythematosus (SLE) [53]. Furthermore, MSCs have been found to improve joint function and reduce the severity of symptoms in horses with chronic autoimmunologic inflammatory diseases [54]. However, while the potential benefits are clear, further research is needed to fully understand the long-term effects and optimize treatment protocols in equine patients. The systemic administration of ESCs is still in the experimental stages but holds promise as a new approach to managing chronic inflammatory diseases in equines.

## 4. Therapeutic Potential in Canine Medicine

Stem cells have been applied to treat various conditions in dogs, including joint diseases, immune-mediated disorders, and degenerative conditions of the heart, kidney, and skin [55,56,57,58,59].

Mesenchymal stem cell therapy for canine osteoarthritis (OA) has garnered significant attention due to its potential for tissue regeneration and reducing pain and inflammation, making it an appealing option for treating OA in dogs. This degenerative joint disease, affecting millions of dogs globally, leads to pain, reduced mobility, and diminished quality of life [60,61,62]. Traditional treatments, such as non-steroidal anti-inflammatory drugs and surgery, often provide only symptomatic relief [63]. MSCs are typically derived from the dog’s adipose tissue or bone marrow. There are no clinical data about using MSCs obtained from the canine uterus. Studies have demonstrated that MSC injections can reduce inflammation, promote cartilage healing, and improve mobility in dogs suffering from OA [64]. For example, a study by Cabon et al. found that dogs treated with MSCs showed significant improvement in clinical signs of OA, including reduced pain and increased activity levels [65]. Additionally, research by Kriston-Pal et al. highlighted that stem cells could modulate the local immune response in OA-affected joints, thereby reducing inflammatory mediators contributing to pain and joint degradation [66]. Moreover, stem cell therapy may offer long-term benefits in OA management. Puzon et al. conducted a study evaluating the long-term effects of MSC injections, finding that the benefits of stem cell therapy persisted for at least 18 months after the initial treatment [67]. This suggests that stem cell therapy may alleviate immediate symptoms and help slow the progression of OA, potentially delaying the need for more invasive procedures such as joint replacement surgery. However, while the early results are promising, more research is necessary to determine the optimal protocols for stem cell therapy in terms of dosage, frequency, and timing of administration. Clinical trials have emphasized the need for standardized treatment regimens to ensure the consistency and reproducibility of results across different populations of dogs [68,69].

Stem cell therapy is revolutionizing the treatment of tendon and ligament injuries in dogs by offering regenerative solutions that address the root cause of damage rather than merely managing symptoms. Mesenchymal stem cells, derived from sources such as adipose tissue, bone marrow, or umbilical cord blood, have demonstrated the ability to modulate inflammation, promote tissue repair, and enhance healing in canine models [70]. Clinical trials provide robust evidence of their efficacy. For example, a randomized study by Cuervo et al. evaluated dogs with tendon injuries treated with autologous adipose-derived stem cells and plasma rich in growth factors, finding significantly faster healing times, reduced lameness, and improved tendon structure compared to conventional therapies [71]. Black et al. demonstrated that MSC therapy reduced pain and enhanced the biomechanical strength of healing tendons, suggesting its potential to restore functional integrity [72]. Further studies reinforce these findings. The following reports documented significant improvements in dogs with cranial cruciate ligament (CCL) injuries treated with allogeneic MSCs, reporting reduced fibrosis and accelerated ligament regeneration without adverse immune responses [73]. Using bone marrow-derived MSCs in tendon repair shows improved collagen organization and reduced scar formation in both canine and equine models [35]. Moreover, a clinical trial by Vilar et al. demonstrated superior long-term mobility and joint health in dogs with soft tissue injuries following intra-articular injections of MSCs compared to corticosteroid treatments [74]. Another study focused on treating partial Achilles tendon tears, showing significant structural and functional improvement after MSC administration [75]. The subsequent research also highlights innovative methods for delivering stem cells. For instance, researchers explored platelet-rich plasma (PRP) as a carrier for MSCs, observing enhanced healing outcomes due to the synergistic effects of growth factors and stem cells [76]. This is supported by studies such as that of Liu et al., which demonstrated that MSCs combined with fibrin-based scaffolds could improve the healing of significant tendon defects [77]. Collectively, these studies emphasize the potential of stem cell therapy to transform soft tissue injury treatment in dogs. However, while clinical outcomes are promising, further large-scale, long-term studies are necessary to refine dosing, delivery methods, and safety profiles.

Dilated Cardiomyopathy (DCM) is a progressive cardiac condition in dogs, marked by ventricular dilation, reduced myocardial contractility, and arrhythmias, often culminating in congestive heart failure and a poor prognosis [78]. In recent years, mesenchymal stem cell therapy has emerged as a promising approach to improve heart function during DCM. MSCs from adipose tissue, bone marrow, or umbilical cord blood possess regenerative and immunomodulatory properties. These cells secrete growth factors such as vascular endothelial growth factor (VEGF) and transforming growth factor-beta (TGF-β), promoting angiogenesis, reducing fibrosis, and enhancing myocardial repair [79,80]. Additionally, dogs receiving autologous MSCs intravenously showed significant improvements in left ventricular ejection fraction (LVEF) and fractional shortening (FS) [81]. Another study highlighted reductions in myocardial fibrosis and arrhythmias and improved systolic function following intracoronary MSC administration in patients with myxomatous mitral valve disease. This study found that dogs in the control group showed a notable decline in heart function, as indicated by a significant increase in left atrial diameter and E-wave velocity on echocardiograms. In contrast, quality-of-life assessments demonstrated improvements in the MSC group, especially in appetite scores, which significantly increased after treatment [82]. Importantly, these studies also indicated that MSC therapy is safe and well-tolerated, with minimal adverse effects. While these findings underscore the potential of stem cell therapy as an adjunct to traditional treatments like ACE inhibitors, beta-blockers, and antiarrhythmics, challenges such as optimizing cell dosage, delivery methods, and long-term efficacy remain. Continued research and more extensive clinical trials are necessary to establish MSC therapy as a viable standard treatment for canine DCM or other heart disorders [83].

Stem cell therapy is gaining traction as a potential treatment for chronic kidney disease (CKD) in dogs, a progressive condition that affects renal function and often leads to severe complications [84]. Mesenchymal stem cells have shown promise due to their anti-inflammatory, immunomodulatory, and regenerative properties, which can mitigate kidney damage and improve renal function [85]. A study by He et al. evaluated the effects of adipose-derived MSCs in dogs with CKD, finding significant improvements in serum creatinine levels, proteinuria, and renal histopathology compared to untreated controls [86]. Additionally, a clinical trial demonstrated that MSC treatment slowed disease progression, as evidenced by reduced fibrosis and improved tubular regeneration [87]. These findings highlight the potential of stem cell therapy as a novel approach to managing CKD in dogs. However, larger-scale studies are needed to optimize dosing strategies and assess long-term safety and efficacy.

Stem cell therapy has shown significant promise in addressing atopic dermatitis (AD) and enhancing wound healing in dogs, offering an innovative approach to managing these conditions [88]. Mesenchymal stem cells possess potent immunomodulatory and anti-inflammatory properties, critical in treating inflammatory skin disorders like AD [89,90,91,92,93]. A clinical trial conducted by Ramos et al. demonstrated that dogs with atopic dermatitis treated with adipose-derived MSCs exhibited marked reductions in pruritus and erythema and long-lasting improvements in overall skin condition compared to placebo-treated controls. Combining these regenerative and anti-inflammatory effects also addresses underlying immune dysregulation, a key factor in atopic dermatitis. Kang et al. demonstrated reduced serum IgE levels and inflammatory cytokines in MSC-treated dogs [94].

The therapeutic benefits of MSCs in wound healing show that dogs treated with MSCs experienced accelerated wound closure, improved angiogenesis, and better-quality tissue regeneration than those treated with traditional methods [89]. These findings underscore the potential of stem cell therapy to revolutionize the management of chronic skin conditions and acute wound healing in veterinary medicine.

Stem cell therapy is emerging as a potential treatment for canine cognitive dysfunction syndrome (CCDS), a neurodegenerative condition in aging dogs that parallels Alzheimer’s disease in humans. Mesenchymal stem cells offer neuroprotective, anti-inflammatory, and regenerative effects, which make them a promising option for managing CCDS [95]. Clinical trials have demonstrated that the administration of autologous skin-derived neural precursor cells (NPCs) in dogs with CCDS improved cognitive function and reduced anxiety-related behaviors in dogs [96]. Another study found that MSCs can reduce brain inflammation and oxidative stress, key contributors to cognitive decline, while promoting human neurogenesis. This may suggest the usefulness of using such therapy in dogs [97]. These findings underscore the potential of stem cell therapy to slow neurodegeneration and improve cognitive and behavioral symptoms in dogs with CCDS. However, large-scale studies are needed to refine protocols, determine optimal dosing, and confirm long-term efficacy.

## 5. Therapeutic Potential in Feline Medicine

Chronic kidney disease is one of the most common conditions in older cats, affecting a significant portion of the aging feline population. Traditional treatment options for CKD in cats, such as dietary changes and medications, aim to slow disease progression but are not curative [98,99,100]. Stem cell therapy has been investigated as a novel approach to this condition. MSCs have shown promise in reducing kidney inflammation and fibrosis, potentially slowing the progression of CKD and improving kidney function. In clinical trials, some cats have exhibited mild improvements in kidney function after intravenous administration of stem cells [101]. In one study, cats with CKD were treated with autologous adipose-derived MSCs. Results showed a reduction in inflammatory markers and a modest improvement in renal function, though the long-term impact of stem cell therapy on CKD progression remains under study [102].

Feline Chronic Gingivostomatitis (FCGS) is a severe, inflammatory condition that can cause significant pain and discomfort in affected cats. Traditional treatments, including corticosteroids, antibiotics, and tooth extractions, often provide limited relief [103,104]. Stem cell therapy offers a new approach to managing this challenging condition. MSCs have immunomodulatory and anti-inflammatory properties that may help reduce the inflammation associated with gingivostomatitis. Clinical trials have reported that cats treated with MSCs experienced a significant reduction in oral inflammation and pain [105,106]. In one clinical study, AD-MSCs were administered systemically to cats with gingivostomatitis, and approximately 70% of cats showed a positive response, with decreased inflammation and improved quality of life [107].

Recent studies have explored mesenchymal stem cells as a novel therapy for feline asthma, a chronic inflammatory disease characterized by airway hyperresponsiveness (AHR), eosinophilic inflammation, and structural airway remodeling. Traditional treatments such as corticosteroids and bronchodilators help manage symptoms but do not reverse airway remodeling [108]. In preclinical studies, MSCs have demonstrated immunomodulatory and anti-inflammatory effects, reducing airway inflammation and fibrosis [109]. A pilot study by Trzil found that the intravenous administration of adipose-derived MSCs in cats with experimentally induced asthma led to significant reductions in computed tomography (CT) measures of airway remodeling. However, airway eosinophilia and AHR remained unchanged [108]. Similar findings in murine models suggest MSCs may influence airway structure but not immune cell infiltration [110]. Additionally, MSCs secrete paracrine factors that modulate immune responses and promote tissue repair, further supporting their therapeutic potential [111]. While MSC therapy appears safe, further research is needed to optimize dosing regimens, evaluate long-term efficacy, and clarify its role alongside conventional asthma treatments.

Feline inflammatory bowel disease (IBD) is a chronic condition marked by persistent inflammation in the gastrointestinal tract, causing symptoms like vomiting, diarrhea, and weight loss [112]. While traditional treatments, including corticosteroids and immunosuppressive drugs, can help manage symptoms, they often fail to address the underlying damage to the mucosal lining and immune system dysregulation [113]. In recent years, mesenchymal stem cells have been proposed as a potential treatment option for IBD in cats due to their ability to modulate inflammation and promote tissue repair. According to Carvalho, MSCs have shown promising anti-inflammatory properties in various conditions, including IBD, by supporting tissue regeneration and helping to restore intestinal health [114]. In a study by Webb and Webb, the use of adipose-derived MSCs in cats with IBD resulted in notable improvements, reducing clinical symptoms and inflammatory cell infiltration in the affected areas [115]. Their findings suggest that MSC therapy could be as effective as standard treatments, particularly in cases where conventional drugs do not provide satisfactory results. Nonetheless, Zhang notes that while these early findings are promising, further research is necessary to determine the most effective dosage and to assess the long-term safety of MSC-based treatments in feline IBD [116]. These insights point to MSC therapy as a potential alternative for managing chronic inflammatory conditions in cats, especially for those who do not respond well to traditional treatments.

## 6. Therapeutic Potential in Bovine Medicine

Mastitis is a serious illness that costs the dairy sector some USD 35 billion annually worldwide [117]. It is a complicated, multi-etiological illness with several microbiological and environmental risk factors [118]. About 150 different bacterial species and subspecies are thought capable of causing mastitis in dairy cattle. Nonetheless, members of just ten groups are responsible for over 95% of the instances. These groupings are separated into infectious or environmental diseases based on their reservoir and transmission mode [119,120]. *Trueperella pyogenes*, *Staphylococcus aureus*, *Streptococcus agalactiae*, *Streptococcus pyogenes*, *Escherichia coli*, *Klebsiella pneumoniae*, *Enterobacter aerogenes*, and *Pasteurella* spp. are the most commonly implicated bacteria [121,122,123]. Most pathogens responsible for infections are *Staphylococcus aureus*, while the main environmental pathogens belong to the *Enterobacteriaceae* family, particularly *Escherichia coli* and *Streptococcus uberis* [124]. According to Cortinhas et al., *Klebsiella* spp. and *E. coli* are the most frequently isolated Gram-negative bacteria in clinical mastitis, whereas *Streptococcus agalactiae* and *S. aureus* are the most common Gram-positive bacteria. Transmission of *S. agalactiae* and *S. aureus* primarily occurs through direct contact [125,126]. Research indicates that approximately 50% of mastitis cases are caused by *S. aureus*, but only 10% to 30% of these cases respond to antibiotic treatment [127,128].

A study by Peralta et al. demonstrated that the administration of MSCs derived from bone marrow and adipose tissue in dairy cows with experimentally induced *Staphylococcus aureus* mastitis led to a decrease in bacterial counts and somatic cell counts in milk, indicating an improvement in udder health [129]. Further supporting the efficacy of MSCs in mastitis treatment, it was concluded that MSC therapy significantly reduced inflammation and promoted tissue regeneration in cows with mastitis [130]. Another investigation found that the intramammary infusion of MSCs reduced clinical symptoms of mastitis and enhanced the immune response in affected cows [131]. Antibiotics are effective in treating bovine mastitis, but they do not address the regeneration of mammary glandular tissue and have been associated with an increase in antimicrobial resistance worldwide [129]. However, MSCs have the capacity for differentiation into mesodermal lineages, including osteogenic, chondrogenic, and adipogenic [130]. This may suggest that in the future, it will be possible to create techniques using MSCs that will enable udder regeneration after mastitis. Still, these days, the main course of MSCs is based on direct interaction with immune cells and local microenvironmental variables to influence the actions of most immune effector cells [132]. These studies collectively suggest that MSC therapy could offer a promising alternative to traditional treatments, reducing reliance on antibiotics and supporting tissue repair.

Cattle are often prone to orthopedic issues, especially in intensive farming settings where high body weights and physical activity can cause musculoskeletal injuries. Stem cell therapy has emerged as an option for treating cartilage injuries, bone fractures, and joint degeneration in cattle. In bone fracture, MSC can be helpful for three main reasons: anti-inflammatory potential, ability to increase angiogenesis, and supportive role in the regeneration of tissue functionality [133,134]. It should be remembered that with orthopedic issues, milk production decreases due to pain, stress, or the inability to feed. The decrease in milk sold is also influenced by the restrictions introduced on animals subject to antibiotic therapy. Therefore, new applications of stem cells in cattle orthopedic injuries should be intensively investigated to minimize losses resulting from reduced milk production associated with fractures. Osteoarthritis is a disease that can significantly reduce fertility in cattle [135]. The leading cause of this disease is the release of pro-inflammatory cytokines [136]. Currently, no studies indicate the possibility of using MSCs to treat OA in cattle. In contrast, numerous experimental and clinical studies have proven the possibility of treating OA using MSCs in horses. Given the ability of MSCs to influence the immune response [137], it can be assumed that there is potential to use MSCs to treat OA in cattle.

Stem cells also have potential applications in in vitro meat production and biopharming. MSCs harvested from bovine muscle tissues are being used to develop lab-grown meat. This method could provide a sustainable alternative to traditional meat production, reducing the environmental impact and addressing ethical concerns regarding animal welfare [138]. MSCs are used in the genetic engineering of transgenic cattle that can produce therapeutic proteins in their milk. These proteins are then harvested for human medicine, representing a cross-species application of stem cell technologies [135].

Cell therapy products for food-producing animals are subject to stricter regulations than those for non-food animals to ensure food safety [139]. Under EU law (Directive 2001/82/EC), only medicinal products that have passed a positive residue assessment or are classified as out-of-scope substances (which pose no known health risks) can be used. Stem cells are currently considered out-of-scope, allowing their use in autologous and allogeneic treatments. However, it is unclear whether this classification applies to differentiated cells (e.g., chondrocytes). While being out-of-scope exempts stem cells from residue assessments, market authorization is still required before they can be used in food-producing animals [140].

## 7. MSCs’ Mechanism of Action

**Figure 2:**
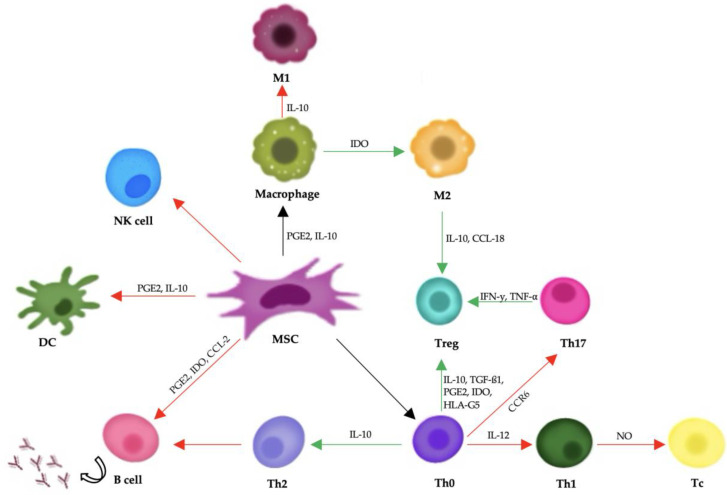
A diagram showing mesenchymal stem cells’ influence on the body’s immune response. Red arrows represent negative impacts, and green arrows represent positive impacts. Abbreviations: PGE2: prostaglandin E2; IFN-γ: interferon-γ; TNF-α: tumor necrosis factor-α; TGF-β1: transforming growth factor-β1; IDO: indoleamine-pyrrole 2,3-dioxygenase; IL: interleukin; PD-1/PD-L1: programmed death-1/programmed death-ligand 1; CCR6: chemokine receptor 6; CCL-2: C-C motif chemokine ligand 2; CCL-18: C-C motif chemokine ligand 18.

Mesenchymal stem cells support tissue repair mainly by releasing bioactive substances through paracrine signaling. They secrete cytokines, growth factors, and extracellular vesicles that help regulate the immune system and encourage regeneration. Important components of this process include transforming growth factor-beta (TGF-β), hepatocyte growth factor (HGF), prostaglandin E_2_ (PGE_2_), and interleukin-10 (IL-10), all of which can reduce inflammation and aid in tissue healing (Figure 2) [141].

In addition to their role in modulating the immune system, MSCs engage with the extracellular matrix and support the formation of new blood vessels (angiogenesis). They produce matrix metalloproteinases (MMPs), which help restructure the extracellular matrix, enabling cell movement and tissue regeneration. Furthermore, vascular endothelial growth factor (VEGF) released by MSCs stimulates angiogenesis, which is essential for re-establishing blood flow to damaged tissues [142].

Mesenchymal stem cells exert their therapeutic effects primarily through immunomodulation and the secretion of soluble factors, rather than by directly differentiating into damaged tissues. They have the ability to interact with various immune cells, including T cells, B cells, dendritic cells, and natural killer (NK) cells. MSCs suppress the activation and proliferation of effector T cells and B cells, inhibit the maturation and antigen-presenting function of dendritic cells, and reduce the cytotoxic activity of NK cells. At the same time, they promote the expansion of regulatory T cells, which play a crucial role in maintaining immune tolerance and suppressing inflammation [141,142,143].

## 8. Conclusions

Analyzing the existing literature and studies on mesenchymal stem cells in veterinary medicine demonstrates their significant potential in regenerative therapy across various animal species. MSC-based therapies have been investigated in treating musculoskeletal disorders, inflammatory conditions, and degenerative diseases, showing promising clinical outcomes. MSC therapy has demonstrated substantial benefits in treating tendon injuries, osteoarthritis, and reproductive disorders in equine medicine. Studies indicate that MSCs improve tissue regeneration and reduce inflammation, facilitating faster recovery and lowering the risk of reinjury in tendonitis cases. Similarly, in osteoarthritis, MSC injections have contributed to cartilage repair, reduced pain, and increased mobility. Canine applications of MSC therapy have proven effective in osteoarthritis, tendon injuries, dilated cardiomyopathy, chronic kidney disease, atopic dermatitis, and cognitive dysfunction syndrome, leading to improvements in mobility, tissue repair, and immune modulation. In feline medicine, MSC therapy has been primarily explored for chronic kidney disease, gingivostomatitis, asthma, and inflammatory bowel disease, where it has shown potential to reduce inflammation and fibrosis, alleviate pain, and improve clinical symptoms. Studies in bovine medicine have focused on mastitis and orthopedic injuries. MSC administration demonstrates reduced bacterial load, decreased somatic cell counts, and enhanced tissue regeneration, presenting a potential alternative to antibiotics.

Additionally, MSC therapy has been explored for its role in bone fracture healing and joint repair, with findings suggesting it may help mitigate economic losses in dairy farming. Despite the encouraging results, challenges such as variability in cell sources, isolation methods, and administration protocols contribute to inconsistent outcomes. Concerns regarding potential adverse effects, such as tumorigenicity and immune response modulation, necessitate further investigation. While preliminary studies suggest MSC safety in veterinary patients, long-term follow-up studies are essential to assess efficacy and potential risks comprehensively. The findings indicate that MSC-based therapies offer significant promise in veterinary medicine, with demonstrated benefits in regenerative applications across multiple species. Their capacity for tissue repair, immunomodulation, and anti-inflammatory action positions MSCs as a valuable tool for treating various conditions. Moreover, MSC-derived extracellular vesicles, which have low immunogenicity and do not carry a risk of tumor development, may be viable substitutes for whole-cell therapies. However, further large-scale, controlled clinical studies are needed to optimize treatment protocols, address safety concerns, and establish standardized guidelines for MSC application in veterinary practice.

## Data Availability

No new data were created or analyzed in this study.
